# Analysis of tissue and circulating microRNA expression during metaplastic transformation of the esophagus

**DOI:** 10.18632/oncotarget.10291

**Published:** 2016-06-25

**Authors:** Daniela Cabibi, Stefano Caruso, Viviana Bazan, Marta Castiglia, Giuseppe Bronte, Sabrina Ingrao, Daniele Fanale, Antonina Cangemi, Valentina Calò, Angela Listì, Lorena Incorvaia, Antonio Galvano, Gianni Pantuso, Eugenio Fiorentino, Sergio Castorina, Antonio Russo

**Affiliations:** ^1^ Department of Science for Promotion of Health and Mother and Child Care, Section of Human Pathology, University of Palermo, 90127 Palermo, Italy; ^2^ Department of Surgical, Oncological and Oral Sciences, Section of Medical Oncology, University of Palermo, 90127 Palermo, Italy; ^3^ Department of Surgical, Oncological and Oral Sciences, Section of Surgical Oncology, University of Palermo, 90127 Palermo, Italy; ^4^ Fondazione Mediterranea “G.B. Morgagni”, Department of Biomedical and Biotechnological Sciences, University of Catania, 95100 Catania, Italy

**Keywords:** microRNA, Barrett's esophagus, columnar-lined oesophagus, esophagitis, metaplasia

## Abstract

Genetic changes involved in the metaplastic progression from squamous esophageal mucosa toward Barrett's metaplasia and adenocarcinoma are almost unknown. Several evidences suggest that some miRNAs are differentially expressed in Barrett's esophagus (BE) and esophageal adenocarcinoma. Among these, miR-143, miR-145, miR-194, miR-203, miR-205, miR-215 appear to have a key role in metaplasia and neoplastic progression. The aim of this study was to analyze deregulated miRNAs in serum and esophageal mucosal tissue biopsies to identify new biomarkers that could be associated with different stages of esophageal disease. Esophageal mucosal tissue biopsies and blood samples were collected and analyzed for BE diagnosis. Quantitative Real-time PCR was used to compare miRNA expression levels in serum and 60 disease/normal-paired tissues from 30 patients diagnosed with esophagitis, columnar-lined oesophagus (CLO) or BE. MiRNA expression analysis showed that miR-143, miR-145, miR-194 and miR-215 levels were significantly higher, while miR-203 and miR-205 were lower in BE tissues compared with their corresponding normal tissues. Esophageal mucosa analysis of patients with CLO and esophagitis showed that these miRNAs were similarly deregulated but to a lesser extent keeping the same trend and CLO appeared as intermediate step between esophagitis and BE. Analysis on circulating miRNA levels confirmed that miR-194 and miR-215 were significantly upregulated in both BE and CLO compared to esophagitis, while miR-143 was significantly upregulated only in the Barrett group. These findings suggest that miRNAs may be involved in neoplastic/metaplastic progression and miRNA analysis might be useful for progression risk prediction as well as for monitoring of BE/CLO patients.

## INTRODUCTION

Barrett's esophagus (BE) is a premalignant condition of the esophagus, defined as displacement of the normal stratified squamous epithelium by specialized columnar lined epithelium. It is a common condition, affecting up to 2 per cent of the adult population. BE often arises as a consequence of mucosal injury from chronic gastro-esophageal reflux disease (GERD) [1–3].

Although, over the years, several authors have tried to better define this condition does not exist today a universally accepted definition of BE. The American College of Gastroenterology (ACG) reported that BE “is confirmed to have intestinal metaplasia by biopsy of the tubular esophagus” [4]. However, the requirement for identification of intestinal metaplasia on biopsy is not demanded by the British Society of Gastroenterology (BSG) [5].

It is broadly accepted that metaplasia with goblet cells is associated with premalignant dysplasia and risk of progression to esophageal adenocarcinoma (EAC) [6]. Intestinal metaplasia is essential for the diagnosis of Barrett's esophagus and enrolment into endoscopic surveillance programs. Simultaneously, columnar metaplasia in the esophagus without intestinal specialized epithelium is defined as “Columnar-lined oesophagus” (CLO) but whether it is a criterion for inclusion in standardized endoscopic surveillance programs is still a matter for debate [4, 7].

Our previous study demonstrated that CLO differs from the true oxyntic-gastric mucosa for positive cytokeratin 7 immunostaining indicative of a reflux damage [8]. In addition, Agnese *et al*. [9] reported that BE and CLO samples were positive for the Aurora kinase A transcript compared to controls, consistently with its overexpression reported in many types of human malignancies [10, 11], but there were no statistically significant quantitative differences in Aurora-A messenger RNA expression between CLO and BE and p53-positive immunostaining.

It is still unclear how the squamous epithelium undergoes its initial metaplastic transformation to a columnar mucosa, and whether CLO is precursor of intestinal metaplasia. Metaplastic transformation appears to be the key event leading to an unstable epithelium which is capable of neoplastic progression and it is unlikely that the presence of goblet cells can be sufficient for malignant risk to EAC [12–14].

In recent years, the role of microRNAs (miRNAs) as potential and ideal biomarkers has been investigated and developed. MiRNA is a new class of small non-coding RNA molecules, about 20–25 nucleotides in length, encoded by cell endogenous genes which have been shown to negatively regulate more than 30% of the coding genes in the human genome via translational repression or mRNA degradation [15–17]. Changes in miRNA expression could be important in the development of BE and its progression. Among them, miR-143, miR-145, miR-194, miR-203, miR-205, miR-215 appear to have a key role in the metaplasia and in neoplastic progression [18–20].

MiR-143, miR-145 and miR-215 are downregulated in EAC. Similar results have been found in colon, gastric and lung cancer [21–23]. These findings suggest that we may define miR-143, miR-145 and miR-215 as tumor suppressors, with loss of expression contributing to the development of esophageal adenocarcinoma. In addition, it has been reported that miR-194 is upregulated in BE and EAC. An increased miRNA-194 expression has been linked to intestinal epithelium differentiation [24]. Similarly, miR-203 and miR-205 were reported highly expressed in normal squamous epithelium compared with columnar epithelia [25].

In addition, several studies have shown that it is possible to evaluate the expression of cell-free miRNAs in different body fluids. Recently, Bus and colleagues performed miRNA expression profiling using plasma from 6 controls, 8 patients with BE and 8 with EAC showing that circulating miRNAs are differentially expressed in BE and EAC [26].

The aim of this study was to analyze deregulated miRNAs in serum and esophageal mucosal tissue biopsies from subjects undergoing upper gastrointestinal endoscopy in order to identify some biomarkers that could be associated with different stages of esophageal disease.

## RESULTS

### Patients

Esophageal mucosal tissue biopsies and blood samples were collected from individuals undergoing upper gastrointestinal endoscopy at Department of Surgical, Oncological and Oral Sciences, University of Palermo.

The 30 patients enrolled in the study, 9 male and 21 female, showed a mean age of 52 years. All patients were Caucasian and 9 of them had a body-mass index higher than 30. Only six patients were regular smokers at the time of interview. Twelve patients were already included in a program of endoscopic surveillance, while 23 had frequent reflux symptoms at the time of endoscopic evaluation (Table 1).

**Table 1:** Patient characteristics

			Histological Diagnosis		
			Barrett'esophagus	CLO	Esophagitis
**Sex**	Male	9	5	2	2
	Female	21	3	10	8
**Age**	< 50	16	0	6	7
	> 50	14	8	6	3
**Race**	White	30	8	12	10
	Black	0	0	0	0
**Body-mass index**	Obesity	9	1	5	3
	Normal weight	21	7	7	7
**Smoking**	Smokers	6	2	3	1
	Non-smokers	24	6	9	9
**Reflux symptoms**	Frequent	23	6	11	6
	Infrequent	7	2	1	4
**Endoscopic surveillance**	Yes	12	4	5	3
	No	18	4	7	7

**Figure 1:**
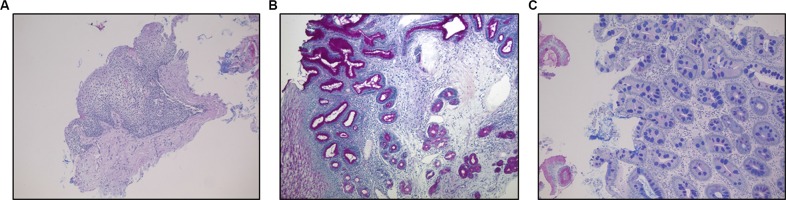
(**A**) Distal esophagitis. (**B**) Non-intestinalized columnar-lined oesophagus (CLO) without acidic mucins and negative for Alcian blue stain; (**C**) Columnar-lined esophageal mucosa with intestinal metaplasia (Barrett's esophagus) characterized by goblet cells appearing as Alcian blue positive cells for the presence of acidic mucins. (A,B,C) Alcian-PAS staining, original magnification 100×.

The histological examination performed on serial sections stained with H & E and Alcian-PAS showed the presence of Barrett's esophagus in 8 patients, CLO in 12 and distal esophagitis (erosive and non) in 10 patients (Figure 1A–1C) (Table 1).

### microRNA expression in esophageal mucosal tissue biopsies

In this study, the expression of miR-143, miR-145, miR-194, miR-203, miR-205 and miR-215 was analyzed in 60 disease/normal-paired FFPE tissue samples used before for histological assessment, from 30 patients diagnosed with esophagitis, CLO or BE (Table 2).

**Table 2:** Mean, median and standard deviation of miR-143, miR-145, miR-194, miR-203, miR-205 and miR-215 relative expression values in relation to the histologic phenotype (Barrett, CLO, Esophagitis)(log scale)

miRNA ID	Barrett (*n* = 8)			CLO (*n* = 12)			Esophagitis (*n* = 10)		
	mean	median	SD	mean	median	SD	mean	median	SD
miR-143	4.55	4.32	1.69	3.13	3.31	1.10	0.68	0.55	1.17
miR-145	4.79	4.59	1.20	3.27	3.34	1.20	0.72	0.57	0.75
miR-194	7.99	8.27	1.31	5.30	6.25	3.16	0.56	0.24	1.74
miR-203	−3.92	−4.32	1.41	−3.09	−3.13	2.59	−0.91	−0.49	1.28
miR-205	−5.62	−5.43	2.97	−4.60	−1.96	5.25	−0.39	−0.27	0.66
miR-215	8.23	8.56	2.33	5.06	5.56	2.53	0.14	−0.20	1.86

The expression miR-143, miR-145 was found upregulated compared to normal tissue of the surrounding esophagus, with a progressive increase during progression from esophagitis to CLO without intestinal metaplasia and then BE with the presence of goblet cells (median fold change > 0.5). The expression of miR-203, miR-205 was significantly downregulated in the group of patients with intestinal metaplasia and to lesser extent in patients with CLO ( median fold change < −0.5) while did not change in patients with esophagitis (median fold change > −0.5).

Conversely, miR-194 and miR-215 were upregulated in patients with BE and with a lesser degree in patients with CLO (median fold change > 0.5) while the expression levels of these miRNAs did not change in patients with esophagitis (median fold change < 0.5).

**Figure 2:**
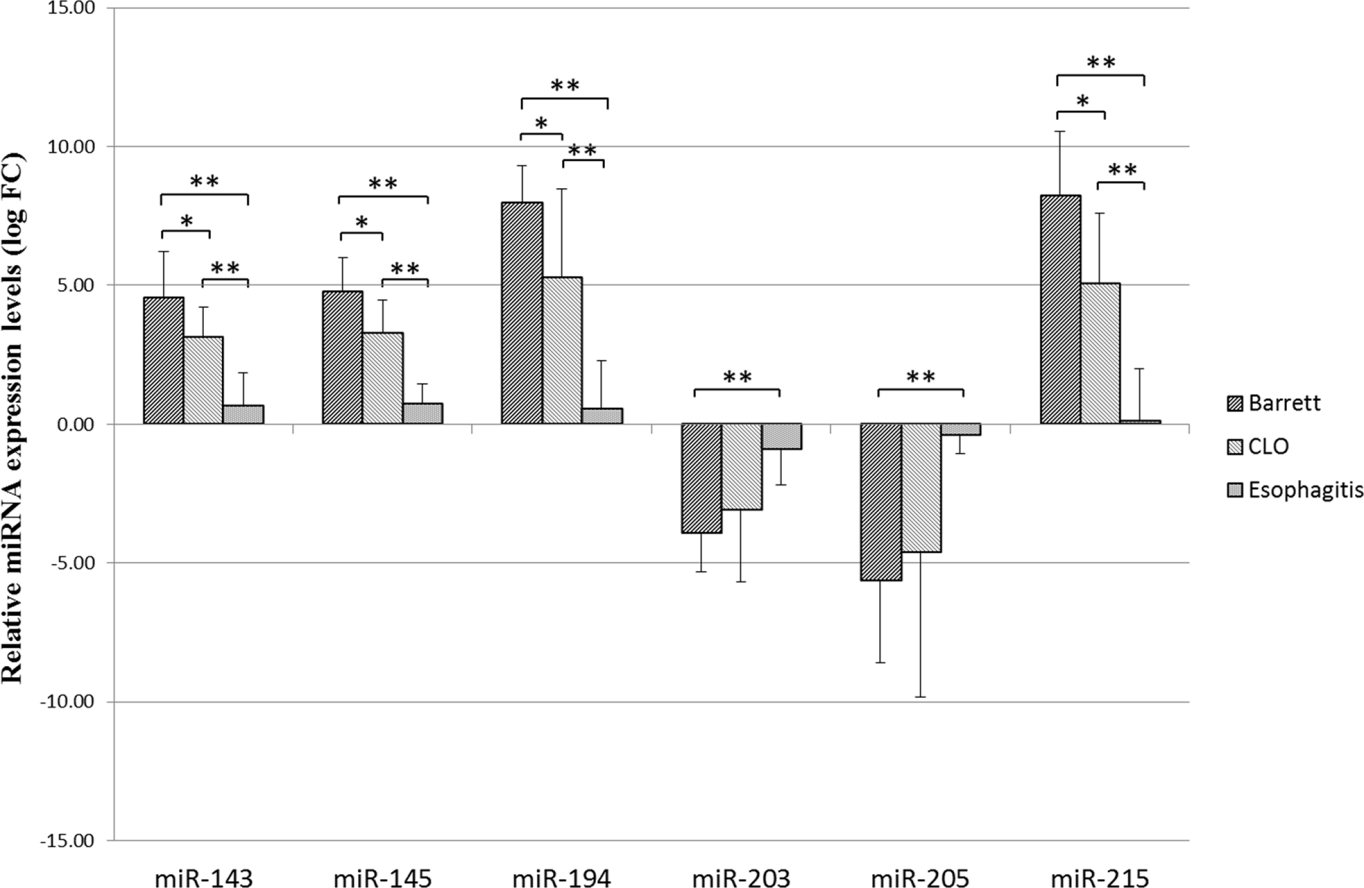
Relative miR-143, miR-145, miR-194, miR-203, miR-205, miR-215 expression levels (logarithmic scale) Relative expression levels are represented as mean values with SD (standard deviation) for each group. Each sample was normalized using the corresponding normal counterpart (10 cm above the Z-line). Normal tissue expression levels were set as 0. Differences in miRNA expression between the different groups were considered significant when the *p* value was under 0.05 (*) and 0.001(**).

Using the Mann Whitney test there were statistically significant differences between the Esophagitis and Barrett groups as regard the expression of all examined miRNAs (Barrett vs Esophagitis). In addition, miR-143, miR-145, miR-194 and miR-215 showed statistically significant differences in expression levels between CLO and Barrett groups (Barrett vs CLO) and between CLO and Esophagitis groups (CLO vs Esophagitis) (Figure 2).

**Figure 3:**
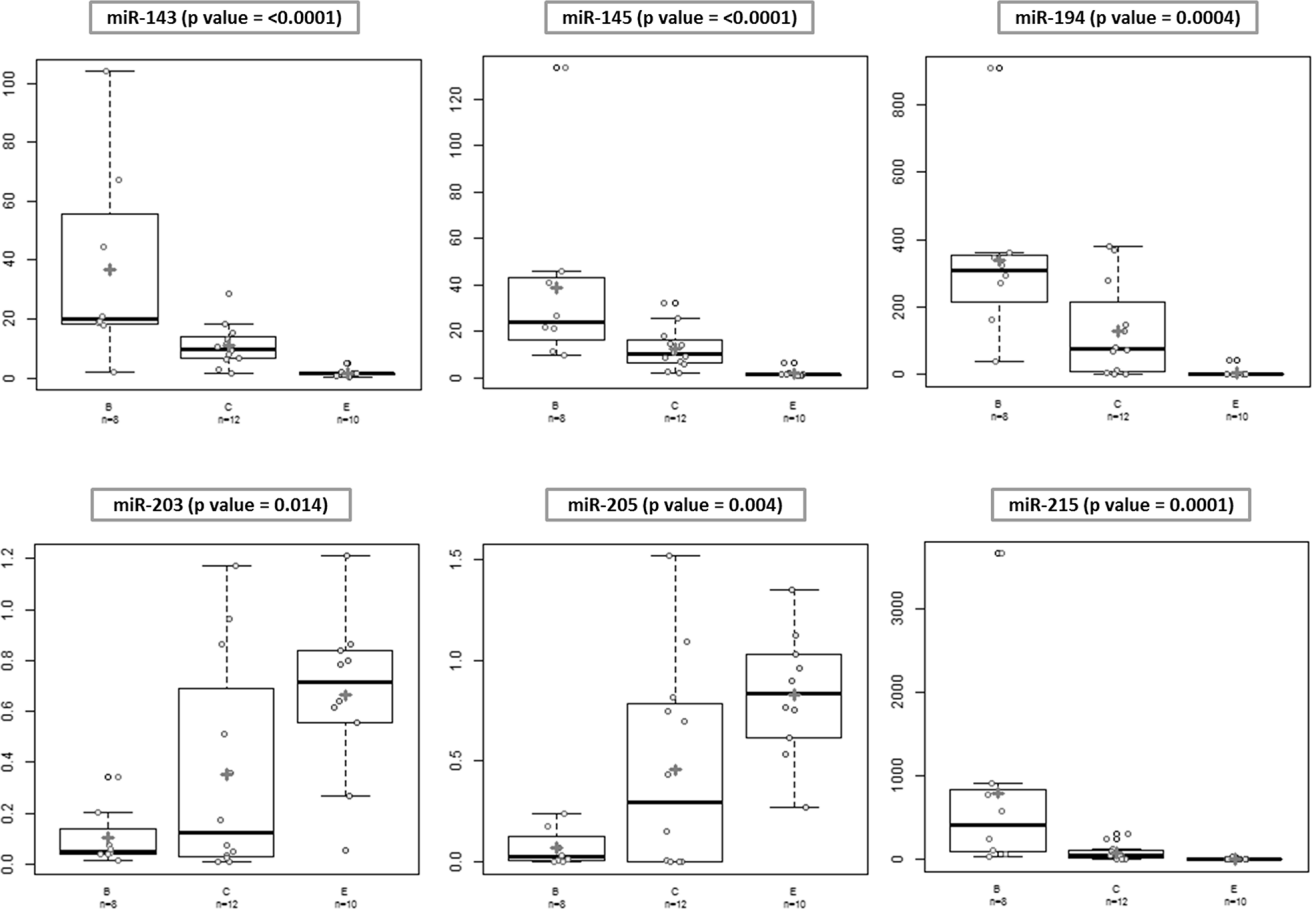
Differences in microRNA expression between different groups Kruskal-Wallis test was used for comparisons of differences in the expression levels in the 3 groups. All the analyzed miRNAs showed statistically significant differences in the expression levels between the groups.

Subsequently, Kruskal-Wallis test that allows the comparison of more than 2 groups, in the present case Barrett vs CLO vs Esophagitis was used. Interestingly, all analyzed miRNAs showed statistically significant differences between the three groups (Figure 3).

### microRNA expression in serum samples

**Figure 4:**
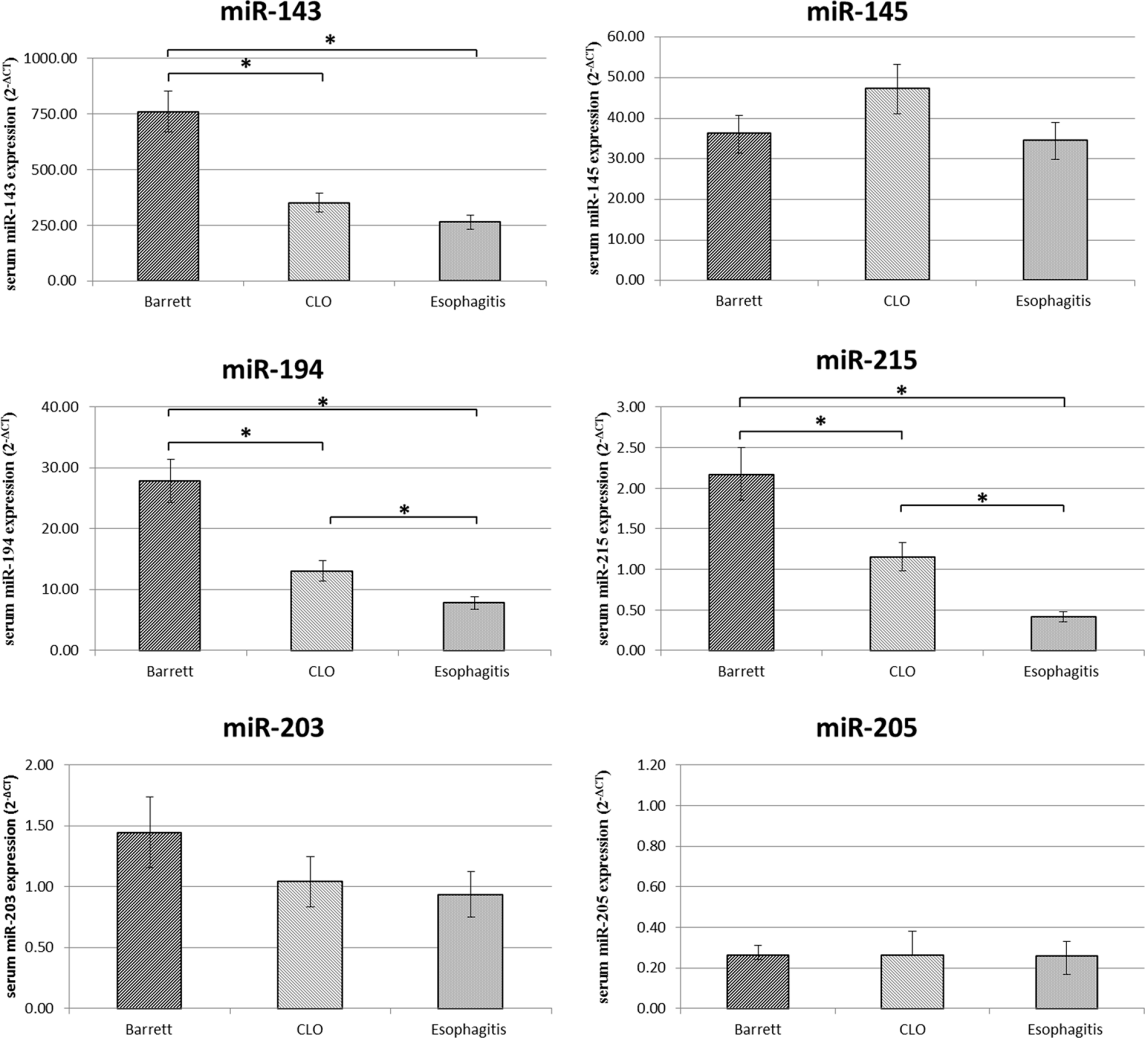
Serum miR-143, miR-145, miR-194, miR-203, miR-205, miR-215 expression levels (2^−ΔCT)^ in Barrett, CLO and Esophagitis groups Differences in miRNA expression between the different groups were considered significant when the *p* value was under 0.05 (*).

Finally miR-143, miR-145, miR-194, miR-203, miR-205, miR-215 serum levels were analyzed in the same patients in order to investigate if there is a relationship with the expression levels in the tissue samples (Figure 4).

Consistent with data from the tissue samples, the expression levels of circulating miR-143, miR-194 and miR-215 were found upregulated in serum of patients with BE compared to patients with CLO and esophagitis. In addition, the miR-194 and miR-215 expression levels were significantly upregulated in the serum of patients with CLO compared to patients with esophagitis reinforcing the hypothesis that CLO is an intermediate step between BE and esophagitis.

However, the expression of serum miR-145, miR-203 and miR-205 not change between the different groups.

## DISCUSSION

MiRNAs are involved in the regulation of a wide range of biological processes, such mechanisms of embryonic development, cell differentiation, apoptosis, cell growth control and regulation of metabolic processes [27–30]. Changes in miRNAs could be important in the development of BE and its progression [18, 31, 32]. Among them, miR-143, miR-145, miR-194, miR-203, miR-205, miR-215 appear to have a key role in the metaplasia and in neoplastic progression. In particular, numerous studies reported the downregulation of miR-143, miR-145, miR-203, miR-205 and miR-215 in EAC [18, 33]. However, few studies have been performed on lesions preceding the adenocarcinoma and in particular on the miRNA expression in the different types of columnar mucosa and esophagitis.

Recent evidence showed that miR-203 and miR-205 are expressed at higher levels in squamous mucosa, and miR-143, miR-145, miR-194 and miR-215 are expressed at higher levels in BE [18].

In this study, it has been shown how the miR-143, miR-145, miR-194, miR-203, miR-205 and miR-215 are differentially expressed in esophageal mucosa with esophagitis, CLO or BE compared to the adjacent normal tissues of the surrounding esophagus. Furthermore, it has been described how these deregulations change between the different groups of patients.

Here, the upregulation of miR-143 and miR-145 was found both in esophagitis than in columnar metaplasia, both gastric and intestinal with a progressive increase during progression from esophagitis to CLO without intestinal metaplasia and then BE with the presence of goblet cells. Similarly, miR-215 was upregulated in patients with BE and with a lesser degree in patients with CLO. These findings are in line with the role that these three miRNAs play in the DNA damage repair process caused by the perpetuation of gastroesophageal reflux. In particular, miR-143, miR-145 and miR-215 are considered tumor suppressors, downregulated in EAC probably due to additional molecular alterations that occur during carcinogenesis, such as loss of p53 homozygosity with consequent alteration of the functionality and ability that p53 exerts on the regulation of these three miRNAs [23, 34–36]. In addition, the analysis of miR-145, most upregulated in BE compared to CLO, is consistent with its role in the differentiation towards the intestinal metaplasia via the feedback circuit with BMP-4, which is reported to be overexpressed in BE [37]. In the present study, miR-145 is also up-regulated in the CLO, although to a lesser extent. In fact, in CLO the intestinal metaplasia is not yet manifested phenotypically, although the presence of some Alcian blue positive cells and the focal positivity for CDX2 suggest that the CLO is an early stage of the same intestinal metaplasia. This hypothesis is confirmed by studying trends in the different groups of all analyzed miRNAs. In fact, although some miRNAs did not show a statistically significant difference in the expression levels between CLO and Barrett and between CLO and Esophagitis, there was a clear trend up or downregulation, under which the CLO appeared as intermediate step between Esophagitis and BE.

miR-194 was found upregulated in patients with BE and with a lesser degree in patients with CLO while the expression levels of this miRNA did not change in patients with esophagitis. An increased miRNA-194 expression has been linked to intestinal epithelium differentiation [24] but this miRNA does not seem to act like *oncomiR* or *anti-oncomiR* and therefore probably the upregulation of miR-194 is maintained even in EAC [18, 38, 39].

The miR-203 was progressively downregulated during progression from esophagitis to CLO and then BE. This downregulation, also found in adenocarcinoma could be related to the loss of native squamous phenotype and emerging columnar morphology [33]. In particular, it has been reported that when miR-203 is lost, p63, a target of miR-203, is continuously expressed in other differentiated suprabasal cells and stimulates their division [40, 41].

The expression of miR-205 was significantly downregulated in the group of patients with BE and to lesser extent in patients with CLO. Previous studies have shown that miR-205 is downregulated EAC [18, 25]. The absence of a statistically significant difference in the expression levels of miR-205 between esophagitis and CLO could indicate that at least for this miRNA there is no significant effect in tumor progression, while the downregulation observed in BE could be in line with the higher risk of neoplastic progression recognized for this lesion.

To date, there are few studies on circulating microRNAs both in BE and EAC. Identifying the correlation between circulating miRNAs and tissue miRNAs would support the hypothesis that circulating miRNAs can serve as ideal biomarkers for various diseases. Here, we reported that miR-194 and miR-215 could be potential non-invasive biomarkers for Barrett's esophagus and probably for CLO. Indeed, consistent with data from the tissue samples, miR-194 and miR-215 were significantly upregulated in BE and CLO compared to esophagitis group, and their expressions appear to increase progressively in serum from esophagitis to CLO and finally to BE. In addition, the expression of circulating miR-143 was found significantly upregulated only in the BE. Our findings suggest that these miRNAs could be used as non-invasive biomarkers of BE and CLO in order to monitor patients at higher risk of developing EAC.

The results of this study confirm that the columnar metaplasia, both gastric-type and intestinal-type, although it is a higher risk for neoplastic progression, still does not have all the molecular alterations that characterize EAC. Further studies in order to re-analyze the expression of these miRNAs in subjects with different degrees of dysplasia and adenocarcinoma will be needed. In our case, considering that it was a prospective study and the low incidence of these cancers, it has not been possible to analyze tissues with this phenotype.

The current endoscopic surveillance programs have several limitations. The endoscopic procedure can be uncomfortable for patients. Many dysplastic or early neoplastic lesions are not visible to the naked eye, resulting in missed lesions on biopsy. Most of patients with BE will not develop EAC, but continue to undergo the anxiety often associated with repeated endoscopies. The most promising improvement of surveillance programs would be risk stratification of patients using a variety of markers, such as clinical risk factors, and biomarkers as miRNAs could have all these features and capabilities.

The upregulation of miR-143, miR-145 and miR-215, as reported in this study, could be considered an increase of the protective mechanism, at least in the earliest stages of the metaplastic process. Since miR-143, miR-145 and miR-215 are downregulated in EAC, further studies could evaluate the expression of these microRNAs in the stages of the metaplastic process in which there is the appearance of dysplasia. Similarly, miR-194, that we found more upregulated in BE compared to CLO, could represent an early biomarker for intestinal metaplasia, currently with a greatest risk for progression to EAC.

In conclusion, these findings suggest that miRNAs may be involved in the neoplastic/metaplastic progression and miRNA analysis might be useful for progression risk prediction as well as for monitoring of BE/CLO patients.

Future efforts should focus on the similarities and differences in the expression of miRNAs between the columnar metaplasia and normal esophageal epithelium, evaluating a greater number of patients with chronic gastroesophageal reflux showing macroscopic changes at the level of the gastroesophageal junction during endoscopic assessment.

## MATERIALS AND METHODS

### Sample collection

Esophageal mucosal tissue biopsies and blood samples were collected from individuals undergoing upper gastrointestinal endoscopy. All participants were provided with complete information about the study and all procedures performed were in accordance with the ethical standards of the institutional and/or national research committee and with the 1964 Helsinki declaration and its later amendments or comparable ethical standards.

A total of 30 pairs of disease tissues and adjacent normal tissues of the surrounding esophagus from 30 patients were obtained prospectively from Department of Surgical, Oncological and Oral Sciences, University of Palermo.

For each patient, the biopsies were collected in neutral buffered formalin at pH 7 for histological evaluation as follows:

- biopsies of healthy esophageal mucosa, taken at 10 cm above the Z-line
- biopsies with endoscopic evidence of esophagitis and/or columnar metaplasia, collected from the esophageal squamous mucosa 2–3 cm above the gastro-esophageal junction.

Blood samples from the same patients were drawn at the time of obtaining peripheral vein access for the endoscopic procedure. Serum samples were isolated by centrifugation (3000 *rcf*) from 5 ml of total blood and stored at −80°C until use.

### Histopathology

The clinical diagnosis was confirmed by histopathology, with Barrett's esophagus defined as the presence of columnar-lined esophageal mucosa with intestinal metaplasia within the distal esophageal segment.

Biopsy samples were formalin-fixed, paraffin-embedded sections (3 μm thick) were obtained and stained with Hematoxylin and Eosin (H & E) followed by special stains for mucins Alcian blue pH 2.5/periodic acid–Schiff diastase staining (Alcian-PAS). All the specimens were reviewed by two different experienced gastrointestinal pathologists before miRNA extraction.

Metaplastic lesions were classified as non-intestinalized or intestinalized based on the presence of goblet cells, assessed by Alcian-PAS staining. Non-intestinalized metaplastic lesions were described here as “columnar lined oesophagus” (CLO).

First, histopathology from the formalin-fixed portion of these tissues was used before miRNA expression analysis, to confirm that the samples consisted of the appropriate epithelium. After, the remaining formalin-fixed, paraffin-embedded tissue was used for gene expression analysis.

### MiRNA isolation

Formalin-fixed, paraffin-embedded biopsy samples were deparaffinized and underwent total RNA and miRNA extraction using miRNeasy FFPE Kit (Qiagen Inc., Valencia, CA, USA) according to the manufacturer's instructions. miRNA yield was determined through a NanoDrop ND-1000 spectrophotometer (CELBIO), and the quality assessed by agarose gel electrophoresis.

Total miRNAs were isolated from thawed serum samples using NucleoSpin miRNA Plasma kit (Macherey-Nagel, Düren, Germany). Total miRNAs were extracted from 300 μL, 600 μL or 900 μL of serum and further eluted in 30 μL of nuclease-free water. The miRNA concentration and quality were assessed with the Bioanalyzer 2100 (Agilent Technologies, CA) using the Agilent Small RNA Analysis kit (Agilent, CA).

### Reverse transcription (RT) and quantitative real-time polymerase chain reaction (qRT-PCR)

Quantitative real-time PCR was used to measure miRNA expression levels in 60 disease/normal-paired esophageal mucosal biopsies and 30 serum samples from the same patients. Ten nanograms of total RNA were reverse transcribed using TaqMan MicroRNA Reverse Transcription Kit (Applied Biosystems, Foster City, CA, USA) according to manufacturer's instructions. RT reactions contained 10 ng of RNA sample, 100 nmol/l stem–loop RT primer, 100 mmol/l deoxynucleoside 5-triphosphates, 50 units/μl MultiscribeTM RT, 20 units/μl RNase inhibitor, 1·5 μl 10 × RT buffer (all from Applied Biosystems) and nuclease-free water. The 15-μl reactions were incubated in a Thermocycler (Eppendorf, North Ryde, New South Wales, Australia) for 30 min at 16°C, 30 min at 42°C, 5 min at 85°C and then held at 4°C.

The following Applied Biosystems assays were used for TaqMan analysis: *hsa-miR-203* (Assay ID 000507); *hsa-miR-205* (Assay ID 000509); *hsa-miR-143* (Assay ID 002249); *hsa-miR-145* (Assay ID 002278); *hsa-miR-194* (Assay ID 000493); *hsa-miR-215* (Assay ID 000518). The reactions were incubated in a 96-well plate at 95°C for 10 min followed by 40 cycles of 95°C for 15 s and 60°C for 1 min. The quantitative PCR was performed on an Applied Biosystems 7900HT fast RT-PCR system, and data were collected and analyzed using ABI SDS version 2.3. Triplicate reactions were performed on all samples. To normalize qRT-PCR reactions, parallel reactions were run on each sample for Small RNA U48 (Assay ID 001006), for both tissue and serum samples. Changes in miRNA expression levels were determined using a comparative CT method.

### Statistical analysis

Data processing and analysis were conducted using tools from Microsoft Excel and Prism GraphPad software (GraphPad Software, CA). Comparisons in the different miRNA expression levels between groups were performed using Mann Whitney or Kruskal-Wallis test depending on the distribution of the data. Only miRNAs with a cut-off of cycle threshold (Ct) < 31 were considered in tissue samples while no cut-off was applied in the case of serum since in this fluid the quantities of starting materials are much lower. Differences in miRNA expression were considered significant when the *P* value was under 0.05 (*) and 0.001(**).
